# Analysis of factors influencing technical efficiency of public district hospitals in KwaZulu-Natal province, South Africa

**DOI:** 10.1186/s12960-022-00777-2

**Published:** 2022-11-22

**Authors:** Tesleem K. Babalola, Hammed O. Ojugbele, Moyad Shahwan, Indres Moodley

**Affiliations:** 1 Program in Public Health, Renaissance School of Medicine, Stony Brook University, Stony Brook, NY USA; 2 Department of Public Administration & Governance, Faculty of Business and Management Sciences, Cape Peninsula University of Technology, Cape Town, South Africa; 3 Centre of Medical and Bio-Allied Health Sciences Research, Ajman University, Ajman, UAE; 4 Department of Public Health Medicine, School of Nursing and Public Health, College of Health Sciences, University of KwaZulu-Natal, Durban, South Africa

**Keywords:** Technical efficiency, Hospitals, Healthcare, Africa

## Abstract

**Background:**

District hospitals are crucial in supporting primary health care and serve as a gateway to more specialist care through a referral system. Majority of South Africans access health care services through the public sector district health system. Given the enormous task assigned to the public district hospital within the country, this study examined factors influencing their technical efficiency.

**Method:**

Data were collected for 38 public district hospitals in KwaZulu-Natal province from 2014/15 to 2016/17. Data envelopment analysis (DEA) was used to determine the technical efficiency of the hospitals, adopting both the constant return to scale (CRS) and variable return to scale (VRS) models. Tobit regression model was used to determine factors related to the technical efficiency of the district hospitals.

**Results:**

This study showed that a significant proportion of the district hospitals were technically inefficient. The Tobit regression model identified catchment population, the proportion of inpatients treated per medical personnel, the proportion of inpatients treated per nursing personnel and expenditure per patient day equivalent as factors influencing technical efficiency of the district hospitals.

**Conclusion:**

Findings from this study suggest that the technical efficiency of the district hospitals can be enhanced through an effective referral system and improved peoples’ health-seeking behaviour. In addition, a standard mix of clinical staff toward efficient service delivery and periodic cost analysis of health services with the view to saving cost and maintaining the quality of health care should be considered.

## Background

Healthcare delivery is defined as any effort, whether in personal health care, public health services through inter-sectorial initiatives focusing primarily on promoting, restoring, or maintaining health [1]. The mission of the National Department of Health (nDoH) of South Africa is consistently improving the health care delivery system by focusing on access, equity, efficiency, quality, and sustainability [2]. However, the history of South Africa has a pronounced effect on the health of the people, health policies and service delivery [3]. Pre-apartheid, political, economic, and land restriction policies structured society according to race, gender, and age-based hierarchies, which greatly influenced the organisation of social life, access to essential resources for health, and health services [3].

The World Bank policy study on financing health services in developing countries identified inefficiency as one of the major problems of African health care systems and the others being resource allocation and inequity [4]. The achievement of national and international health development targets requires not only an increase in funding but also the efficient use of available resources and greater equity in financing and accessing quality health care [5, 6]. Health facilities are deemed efficient if they can produce the maximum possible output for a given amount of input [5]. The provision of health services relates to having an appropriate health workforce in terms of numbers, the quality of skills they possess, how as well as where they are deployed, and how they are managed [7]. A recent review of the healthcare facilities assessment in sub-Saharan Africa (SSA) showed a reported below-average efficiency performance of health facilities in different regions of SSA [8].

Technical efficiency is concerned with generating maximum outputs with the least possible input [9]. Assessing the effectiveness of the health care system may assist in determining, by a given standard, which organizations manage their resources and procedures optimally to achieve ideal production levels [10]. Universal health coverage requires a high level of health service output through access to an essential set of health interventions for those in need. Achieving this coverage requires adequate use of health resources efficiently. Efficiency in the health sector is about attaining the highest level of health possible using the available resources. Inefficiency relating to health care service delivery could be linked to different sources. A world health organisation (WHO) report stated that sources of inefficient health care service delivery include sub-optimal quality of care and medical error that could be associated with insufficient guidelines, standards or protocols; poor coordination; and inadequate supervision [11]. It also identified inappropriate hospital size, which could be as a result of uneven historical development of hospitals; inadequate planning, coordination and control as another source [11]. Finally, the report linked inefficiency in health care services to inappropriate hospital admissions or length of stay, which could be due to lack of alternative care plans; insufficient incentives to discharge; and limited knowledge of best practice [11].

South Africa has an estimated population of 55 million people with KwaZulu-Natal province, accounting for about 20% of the population [7, 12]. The health care system comprised of  public sector financed through government funding and a private system funded through health insurance and out of pocket payments [13]. The public health facilities of the country are organised into primary healthcare clinics, district hospitals, regional hospitals, tertiary hospitals, central hospitals, and specialised hospitals [14]. The district hospitals (DHs) forms a significant part of the district health system [15]. The DHs are comprised of small hospitals with 50 to 150 beds, medium-size hospitals with 150 beds to 300 beds, and large hospitals with 300–600 beds [14]. District hospitals play a crucial role in supporting primary health care and serve as a gateway to more specialist care through a referral system [15]. They provide health care services that include inpatient, outpatient and ambulatory health services as well as emergency health services [14].

Since the Abuja declaration of 2001, there has been a steady increase in the health budget of a significant number of countries in Africa [16]. Concurrently, a growing number of studies on health facilities efficiency aimed at identifying and reducing wastage of scarce health system resources were conducted [17]. There have been few of such studies conducted in sub-Saharan Africa (SSA) in the last two decades [17, 18], and a limited number of them examined factors associated with the technical efficiency of health facilities [5, 18–23]. However, not so much has been done to assess the determinants of technical efficiency among health facilities across countries in the southern Africa region [8]. A literature search showed that the latest study that evaluated the causal-effect relationship between technical efficiency scores of health facilities and some explanatory variables was conducted in the year 2001 [24]. Thus, this study was aimed at determining factors associated with the technical efficiency of public district hospitals in KwaZulu-Natal province, South Africa. This study would add to existing knowledge on the efficiency of health facilities in the Southern Africa region and SSA.

## Methods

This is a health system research study involving technical efficiency analysis of 38 public district hospitals in the KwaZulu-Natal province of South Africa. These district hospitals provide generalist health care services to inpatients and outpatients referred from primary and community health centres.

Data envelopment analysis (DEA) approach was used to determine the technical efficiency of the DHs using both the constant return to scale (CRS) and variable return to scale (VRS) models. On the other hand, the Tobit regression model was used to determine factors related to technical efficiency scores of the DHs. DEA measures the ability of health facilities to produce a given level of output using the minimum amount of input or producing the maximum amount of output for a given amount of input [5]. The DEA technique is represented by the following equations: [5, 25, 26]

1 $$\text{Max}\;{E}_{0}=\frac{{\sum}_{r=1}^{s}{u}_{r}{y}_{rj_0}}{{\sum}_{i=1}^{m}{v}_{r}{x}_{ij_0}}$$

Subjected to

2 $$\text{Max}\;{E}_{0}=\frac{{\sum}_{r=1}^{s}{u}_{r}{y}_{rj_0}}{{\sum}_{i=1}^{m}{v}_{r}{x}_{ij_0}} \le 1,$$

$$j=1,\dots {j}_{0}\dots n,\;{u}_{r }\ge {O}_{r}=1, \dots, s\; \text{and}\;{v}_{i }\ge 0,\;i=1, \dots M$$

where *E*_0_ is the technical efficiency; *y*_*rj*_ is the the amount of output *r* from hospital *j*; *x*_*ij*_ is the amount of input *i* to hospital *j*; *u*_*r*_ is the weight given to output *r*, *v*_*i*_ is the weight given to input *i*; *n* is the number of hospital; *s* is the number of outputs; *m* is the number of inputs.

A checklist was developed based on information obtained from the review of previous literature [17, 26, 27]. This checklist together with the information contained in the district hospitals services package [15] was used as a guide for retrieving input, output, and exploratory data for the DHs from the national district health information system (DHIS) database, personnel salary system (PERSAL), and Basic Accounting System (BAS) for three consecutive fiscal years (2014/15, 2015/16 and 2016/17). The input variable used in the DEA efficiency analysis were: medical and dental personnel, nursing personnel; pharmacy personnel, allied healthcare personnel (laboratory scientist/technicians, radiographers, physiotherapists), support and other services personnel (social workers, cleaners, maintenance, security.) and the number of beds. On the other hand, the output variables include total inpatient days, total outpatient headcount, total theatre cases, X-rays done, delivery by caesarean and regular delivery. Finally, the independent explanatory variables were: catchment population, size of the hospital, location of the hospital, average length of stay, inpatient bed utilisation rate, the proportion of outpatients treated by medical personnel, and proportion of inpatients treated by medical personnel. The remaining exploratory variables involved were: the proportion of outpatients attended to by nursing personnel, the proportion of inpatients treated by nursing personnel, outpatient visits as a proportion of inpatient days, the ratio of beds to medical personnel, the ratio of beds to nursing personnel, and expenditure per patient day.

The technical efficiency of the hospitals was estimated using performance improvement management software (PIM-DEA) developed by Thanssoulis and Emrouznejad [28] and data envelopment analysis program (DEAP) by Coelli [29]. DHs that assumed the “best practice frontier” were assigned an efficiency score of “1” or “100%” and are said to be technically efficient compared to others. DHs below the efficiency frontier that scored between “0” and “1” were said to be inefficient. The technical efficiency score was estimated under the CRS and VRS technical efficiency models. The CRS model measures technical efficiency with the assumption that an increase in the input will lead to a proportional increase in the level of output, while the VRS model assumes that an increase in the level of input with either increase or decrease the level of output [25, 30].

The Tobit regression model analysis was employed in determining factors influencing the technical efficiency of the DHs. It was recommended by a comparative study [31] of the appropriateness of different techniques in modeling DEA efficiency scores against independent variables. It was said to be better fitted and sufficient when compared with other techniques such as Ordinary Least Square (OLS), the Papke–Wooldridge (PW) model, and the unit inflated beta model [31]. Considering the appropriateness and the score limit of 0 and 1 for technical efficiency, the Tobit regression model was adopted in this study to relate the CRS and VRS technical inefficiency scores to the independent explanatory variables. The Tobit model was computed by assuming a censoring point at zero so that efficient facilities are constrained at zero while the inefficient facilities assumed scores greater than zero [4, 32]. The CRS and VRS technical efficiency scores were transformed to inefficiency scores and were left-censored at “0” and right-censored at “1” as stated in the equation below:

3 $$\left[\text{Inefficiency}\;\text{score }=\frac{1}{\text{TE}\; \text{score}}\right]-1$$

The transformed CRS and VRS inefficiency scores were regressed separately against exploratory variables. The Tobit regression model is represented below:

4 $$\begin{aligned} {\text{Ineff}}. & = \beta_{0} + \beta_{1} {\text{CatP}} + \beta_{2} {\text{L}}.{\text{fac}}. + \beta_{3} {\text{Loc}}. + \beta_{4} {\text{Inpbed}} + \beta_{5} {\text{Outdoc}} + \beta_{6} {\text{Inpdoc}} + \beta_{7} {\text{BedDoc}} \hfill \\ & \quad + \beta_{8} {\text{Outnur}} + \beta_{9} {\text{Inpnur}} + \beta_{10} {\text{BedNurse}} + \beta_{11} {\text{ALoS}} + \beta_{12} {\text{Outinp}} + \beta_{13} {\text{Exp}}./{\text{PDE}} + \beta_{14} {\text{OPDnRef}} + \varepsilon_{i} \hfill \\ \end{aligned}$$

where “Ineff” = inefficiency scores; “CatP” = catchment population been served by the hospital (1 = DHs with catchment population less than or equal to 100 000; 2 = DHs with catchment population between 101 000 and 200 000; 3 = DHs with catchment population above 200 000). “L.fac.”** = ** level or size of facility measured in proxy by the number of bed (1 = small DHs with 50 to 150 beds, 2 = medium size DHs with > 150 to 300 beds, and 3 = large DHs with > 300 beds to 600 beds) [14]. “Loc.” =  location/population been served by the hospital which was categorised based on demographic profile reported by the department of human settlements of KwaZulu-Natal province [33] (0 = rural; 1 = urban).

Other continuous variables included in the model were:

*“*Alos” = Average length of stay measured in days; “Inpbed” = Inpatient bed utilisation rate, “Outdoc” = proportion of outpatients treated by medical personnel, and “Inpdoc” = proportion of inpatients treated by medical personnel. In addition, include are: “Outnur” = proportion of outpatients attended to by nursing personnel, “Inpnur” = proportion of inpatients treated by nursing personnel, and “Outinp” = Outpatient visits as a proportion of inpatient days. Lastly, “BedDoc” = ratio of beds to a medical personnel; and “BedNurse” = ratio of beds to nursing personnel; “Exp/PDE” = expenditure per patient day equivalent (which refers to the average cost per patient per day treated at the DHs); and OPDnRef = rate of not referred OPD new clients. Whereas, *α*_0_, *β*_1_, *β*_2_, *β*_3_, *β*_4_, *β*_5_, … *β*_14_ are coefficients to be estimated, and *ε*_*i*_ is the random error. Tobit regression analysis was conducted to relate the technical inefficiency scores to the proposed explanatory variables using STATA version 15.0 statistical software. The year 2016/17, the last year in the efficiency analysis trend, was used as the reference point.

## Results

**Fig. 1:**
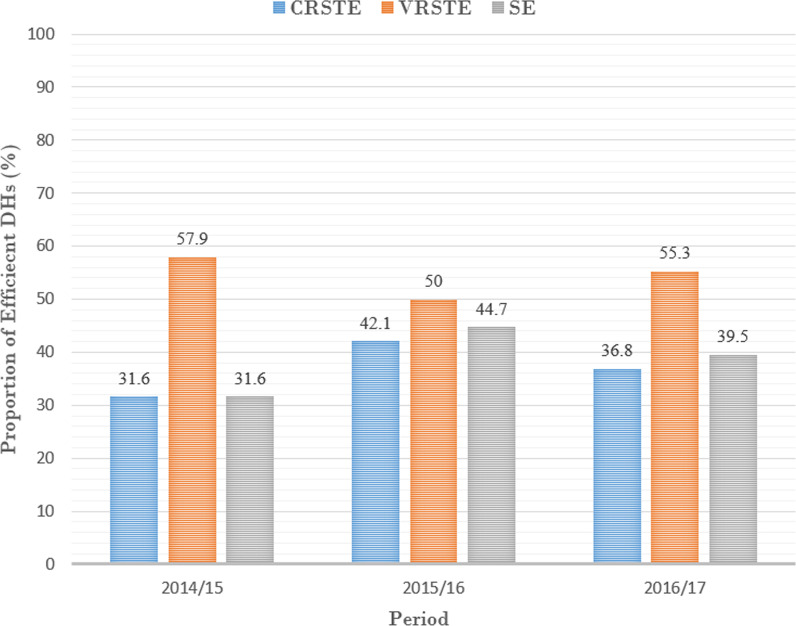
Distribution of technically efficient district hospitals from 2014/15 to 2016/17

The descriptive statistics (the mean, standard deviation, maximum and minimum values) of the input, output and exploratory variables used in the efficiency analysis are shown in Table 1. The proportion of technically efficient DHs for the 3 consecutive years is shown in Fig. 1.

**Table 1:** Descriptive statistics of input, output and exploratory variables

Variable	2014/15	2015/16	2016/17
	Mean (SD)	Mean (SD)	Mean (SD)
Input variables			
Medical and dental personnel	28.68 (28.67)	22.34 (24.88)	21.08 (24.53)
Nursing personnel	407.29 (181.08)	355.03 (164.58)	346.47 (155.84)
Pharmacy personnel	18.11 (9.47)	18.42 (9.82)	18.39 (8.62)
Allied personnel (therapists and Lab. scientists)	22.87 (14.67)	21.34 (13.43)	21.11 (13.57)
Support and other services personnel	271.32 (89.18)	249.50 (84.02)	239.66 (79.63)
Beds	225.47 (87.50)	225.45 (86.70)	229.11 (114.47)
Output variables			
Inpatient days	51 590.26 (24 416.75)	49 485.89 (23 872.68)	45 936.84 (22 734.31)
OPD head counts	62 833.74 (37 418.35)	60 340.39 (36 193.29)	56 545.55 (35 366.38)
Theatre cases	1430.11 (1389.51)	1306.18 (1340.65)	1265.63 (1279.71)
X-ray done	12 284.74 (10 057.77)	12 217.18 (9720.85)	11 819.63 (9580.52)
Delivery by caesarean	651.37 (437.75)	643.50 (427.25)	624.34 (400.50)
Regular delivery	1690.97 (924.42)	1599.00 (904.70)	1560.79 (879.44)
Explanatory variables			
Catchment population	126 987.42 (137 819.20)	126 987.42 (137 819.20)	237 610.68 (225 729.29)
Proportion of outpatient visits to doctor	2651.30 (1293.31)	3374.79 (1499.47)	3417.53 (1431.48)
Proportion of inpatient treated to doctor	2176.61 (877.77)	2830.50 (1202.74)	2902.37 (1305.37)
Bed to doctor ratio	9.80 (4.07)	13.39 (5.79)	14.29 (5.83)
Proportion of outpatient visits to nurse	170.16 (94.82)	187.61 (103.53)	178.20 (96.09)
Proportion of inpatient treated to nurse	135.46 (56.62)	150.41 (64.58)	140.20 (51.35)
Beds to nurse ratio	0.60 (0.20)	0.70 (0.30)	0.68 (0.22)
Average length of stay (days)	5.95 (0.95)	5.92 (0.95)	5.65 (1.03)
Inpatient bed utilization rate (%)	62.08 (11.78)	58.88 (12.02)	56.45 (12.75)
Proportion of outpatient visits to inpatient days	1.33 (0.80)	1.29 (0.61)	1.26 (0.48)
Expenditure per patient day equivalent	2933.64 (816.65)	3268.68 (662.50)	3969.92 (923.61)
OPD not referred	13 000.13 (10 780.01)	11 797.84 (10 035.66)	11 449.34 (9098.31)

Table 2 presents the descriptive statistics of CRS and VRS technical efficiency of the district hospitals and their ranking. The descriptive analysis of technical efficiency scores was closely related for 3 years, with an average mean score ranging from 82.5 to 93.8%. In 2014/15, the mean CRS technical efficiency score was 85.1% with a standard deviation (SD) of 14.0%, the mean VRS technical efficiency score was 90.7% with SD of 12.6%, and the mean scale efficiency score was 93.8% with SD of 7.6%. The mean CRSTE, VRSTE and Scale efficiency scores were 87.4% (SD =  ± 13.7%), 91.8% (SD =  ± 12.0%), and 95.1% (SD =  ± 6.9%) respectively in 2015/16. Finally, the mean CRS and VRS technical efficiency scores were 82.5% (SD =  ± 16.8%) and 90.0% (SD =  ± 13.3%), respectively, in 2016/17, while the mean scale efficiency score was 91.2% (SD ± 9.2%).

**Table 2:** Descriptive statistics of technical and scale efficiency and hospital ranking from 2014/15 to 2016/17

DH ranking (based on DEA scores)	2014/15			2015/16			2016/17		
	CRSTE	VRSTE	SE	CRSTE	VRSTE	SE	CRSTE	VRSTE	SE
	*n* (%)	*n* (%)	*n* (%)	*n* (%)	*n* (%)	*n* (%)	*n* (%)	*n* (%)	*n* (%)
100%	12 (31.6)	22 (57.9)	12 (31.6)	16 (42.1)	19 (50.0)	17 (44.7)	14 (36.8)	21 (55.3)	15 (39.5)
80.0–99.9%	12 (31.6)	7 (18.4)	25 (65.8)	11 (28.9)	12 (31.6)	18 (47.4)	7 (18.4)	9 (23.7)	18 (47.4)
60.0–79.9%	12 (31.6)	7 (18.4)	1 (2.6)	10 (26.3)	6 (15.8)	3 (7.9)	14 (36.8)	7 (18.4)	5 (13.2)
40.0–59.9%	2 (5.3)	2 (5.3)	0 (0.0)	1 (2.6)	1 (2.6)	0 (0.0)	2 (5.3)	1 (2.6)	0 (0.0)
< 40.0%	0 (0.0)	0 (0.0)	0 (0.0)	0 (0.0)	0 (0.0)	0 (0.0)	1 (2.6)	0 (0.0)	0 (0.0)
Mean	0.851	0.907	0.938	0.874	0.918	0.951	0.825	0.900	0.912
SD	0.140	0.126	0.076	0.137	0.120	0.069	0.168	0.133	0.092
Maximum	1.000	1.000	1.000	1.000	1.000	1.000	1.000	1.000	1.000
Minimum	0.537	0.565	0.647	0.568	0.571	0.772	0.378	0.499	0.705

To show the relationship between expected factors influencing technical efficiency (TE), a regression analysis of the transformed efficiency scores and identified explanatory variables was done. The regression analysis results relating the transformed CRS and VRS technical efficiency scores to the explanatory variables are presented in Tables 3 and 4.

**Table 3:** Regression of CRS technical inefficiency scores against exploratory variables

Variables	Coefficient	Std. Err.	*Z*	*P*-value	95% CI	
					Lower limit	Upper limit
Model 1 (CRSTE)						
Catchment population (ref: "≤100,000")						
100 001–200 000	0.258903	0.144427	1.79	0.087	− 0.04062	0.558426
> 200 000	0.093021	0.150426	0.62	0.543	− 0.21894	0.404985
Level of facility (ref: Small "50–150 beds")						
Medium (150–300 beds)	− 0.26425	0.142891	− 1.85	0.078	− 0.56059	0.032083
Large (300–600 beds)	0.11657	0.177804	0.66	0.519	− 0.48531	0.252173
Location	0.017687	0.187435	0.09	0.926	− 0.37103	0.406403
Outpatient/doctor	0.000224	0.000135	1.65	0.113	− 5.7E−05	0.000504
Inpatient/doctor	− 0.00068	0.000252	− 2.69	0.013*	− 0.0012	− 0.00015
Ratio of beds to doctor	0.070006	0.045213	1.55	0.136	− 0.02376	0.163772
Outpatient/nurse	− 0.00981	0.004968	− 1.97	0.061	− 0.02011	0.000493
Inpatient/nurse	0.008444	0.007086	1.19	0.246	− 0.00625	0.02314
Ratio of beds to nurse	− 0.8223	1.188997	− 0.69	0.496	− 3.28813	1.643527
Average length of stay	0.027841	0.040708	0.68	0.501	− 0.05658	0.112263
Inpatient bed utilization rate	− 0.00191	0.003553	− 0.54	0.596	− 0.00928	0.005457
Outpatient/inpatient days	0.231625	0.613416	0.38	0.709	− 1.04052	1.503772
Exp. per PDE	− 0.0003	0.000123	− 2.44	0.023*	− 0.00056	− 4.5E−05
OPD not referred	− 6.30E−06	5.59E-06	− 1.13	0.272	− 1.8E−05	5.29E−06
Cons.	2.33511	1.187342	1.97	0.062	− 0.12729	4.797507
Sigma	0.040635	0.012537			0.02143	0.077053
Number of observations = 38						
Log likelihood = − 0.8818						
Chi-square (*χ*^2^) = 48.82						
*P*-value = 0.0001						

*significant at *P* < 0.05

**Table 4:** Regression of VRS technical inefficiency scores against exploratory variables

Variables	Coefficient	Std. Err.	*Z*	*P*-value	95% CI	
					Lower limit	Upper limit
Model 2 (VRSTE)						
Catchment population (ref: "≤100,000")						
100 001–200 000	0.309614	0.131143	2.36	0.028*	0.03764	0.581588
> 200 000	− 0.099347	0.140021	0.71	0.485	− 0.19104	0.389733
Level of facility (ref: Small "50–150 beds")						
Medium (150–300 beds)	− 0.01381	0.136055	− 0.10	0.920	− 0.29597	0.268352
Large (300–600 beds)	− 0.11933	0.181451	− 0.66	0.518	− 0.49564	0.256976
Location	− 0.07142	0.180832	− 0.39	0.697	− 0.44645	0.3036
Outpatient/doctor	0.000267	0.00016	1.67	0.108	− 6.4E−05	0.000599
Inpatient/doctor	− 0.00095	0.000372	− 2.54	0.019*	− 0.00172	− 0.00017
Ratio of beds to doctor	0.108536	0.064882	1.67	0.109	− 0.02602	0.243092
Outpatient/nurse	− 0.01254	0.00674	− 1.86	0.076	− 0.02652	0.00144
Inpatient/nurse	0.018188	0.008614	2.11	0.046*	0.000325	0.036052
Ratio of beds to nurse	− 1.68278	1.768888	− 0.95	0.352	− 5.35123	1.985666
Average length of stay	0.030973	0.035937	0.86	0.398	− 0.04356	0.105502
Inpatient bed utilization rate	0.000633	0.003667	0.17	0.865	− 0.00697	0.008238
Outpatient/inpatient days	0.684321	0.719553	0.95	0.352	− 0.80794	2.176581
Exp. per PDE	− 0.00018	0.000132	− 1.35	0.190	− 0.00045	0.000095
OPD not referred	− 6.36E−06	5.28E−06	− 1.2	0.241	− 1.7E−05	4.59E−06
Cons.	0.592859	1.129556	0.52	0.605	− 1.7497	2.935415
Sigma	0.029257	0.011305			0.013128	0.065201
Number of observations = 38						
Log likelihood = − 1.0199						
Chi-square (*χ*^2^) = 46.18						
*P*-value = 0.0001						

*significant at *P* < 0.05

Under the CRSTE model, the proportion of inpatients treated to a medical doctor (*p* = 0.013) and expenditure per patient day equivalent (*p* = 0.023) had a negative statistically significant relationship with technical inefficiency scores of the DHs. The relationship indicates that a unit increase in the inpatients treated per medical doctor would decrease the expected inefficiency score by 0.068%, and an increase in the average cost per patient per day seen at DHs would decrease the hospital inefficiency score by 0.03%.

Under the VRSTE model, DHs with a catchment population between 100 001 and 200 000 (*P* = 0.028) and proportion of inpatient to nurse (*P* = 0.046) had a significant positive relationship with the expected hospital inefficiency scores. In contrast, the proportion of inpatients to medical doctors (*P* = 0.019) had a significant negative relationship with the hospital inefficiency scores.

**Fig. 2:**
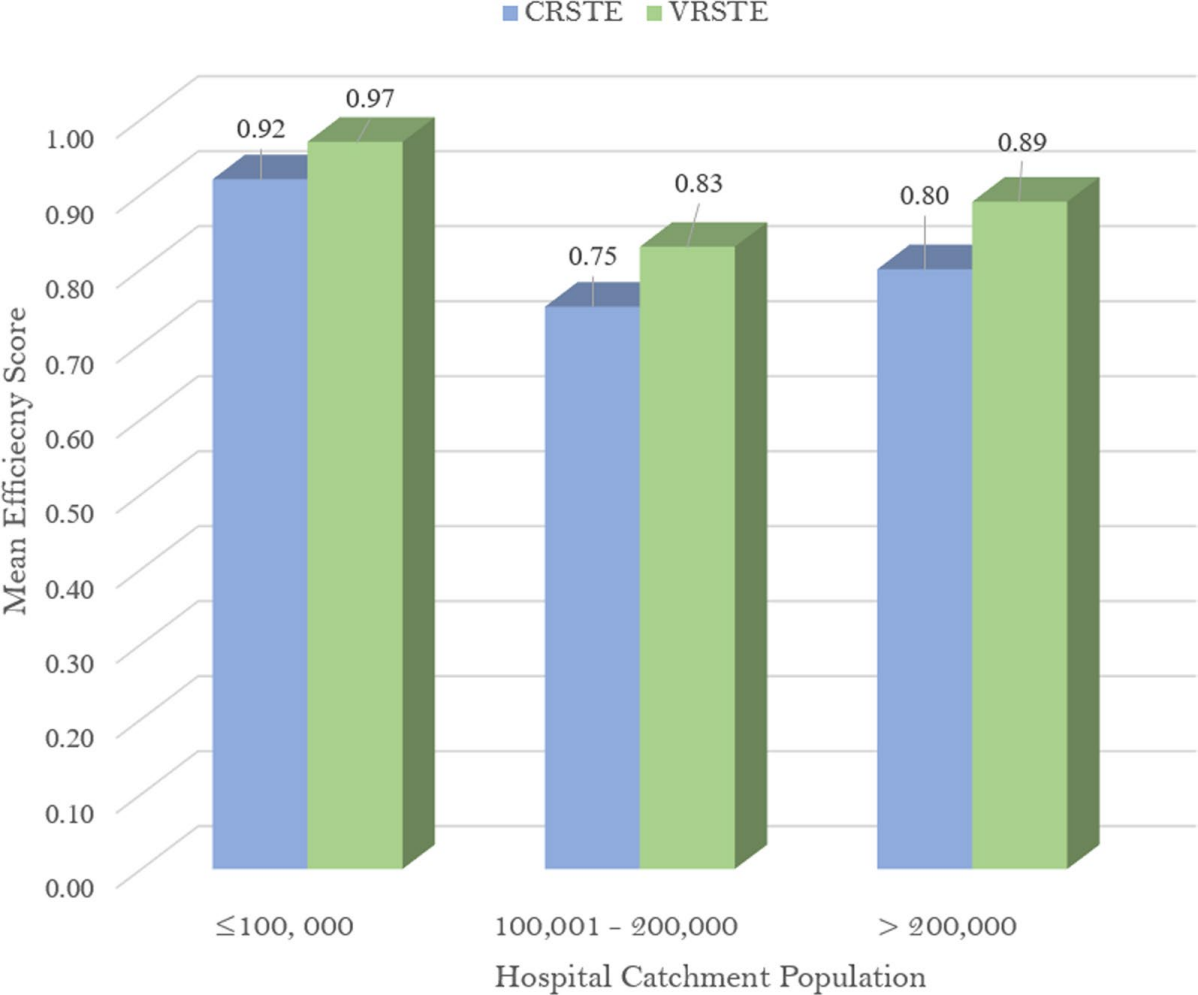
CRS and VRS technical efficiency based on the catchment population

Using DHs with catchment population less than or equal to 100 000 as a reference point, the significant positive coefficient score of DHs with a targeted population between 100 001 and 200 000 as shown in Table 4 suggest that they were technically inefficiency when compared to DHs with a catchment population less than or equal 100 000. As shown in Fig. 2, the mean technical efficiency scores of DHs with less than or equal to 100 000 were higher than those of DHs serving a larger population above 100 000. The significant positive relationship between the ratio of inpatient to nurse and the inefficiency scores indicate that a unit rise in the ratio of inpatient treated to nursing personnel would lead to an increase in technical inefficiency by 1.8%. Similar to the CRS model, the significant negative relationship between the proportion of inpatient to medical personnel and the inefficiency scores signifies that a percentage increase in the ratio of inpatients treated per medical doctor would lead to a decline in the expected hospital inefficiency score by 0.095%.

## Discussion

The findings of this study showed that a significant proportion of public district hospitals studied were technically inefficient during the 3 years. Expenditure per patient day equivalent was significantly related to inefficiency scores under the CRS model. At the same time, the catchment population (100 001–200 000) and the proportion of inpatients treated to a nurse were significant under the VRS model. The proportion of inpatients treated per medical personnel was significantly related to technical inefficiency under both CRS and VRS models.

The significant positive relationship between the catchment population and the technical inefficiency score indicates that DHs serving less than 100 000 catchment population had better technical efficiency when compared with those serving large populations. A similar finding was reported in the assessment of public hospitals in one of the middle eastern countries [34]. The possible explanation for this could be that the DHs with larger catchment population has been stretched and were delivering health services beyond their coping capacity. Therefore, there is a need to look into reducing the burden on these hospitals. The burden can be reduced through strengthening other levels of care, most notably primary health care to meet the growing health demands of the people [35]. Second, the referral system should be improved to control the population of not referred patients seeking care at the DHs who could have visited the primary health care facilities. Another measure that can be taken is to regulate the movement of people seeking healthcare through awareness and ensuring that patients seek care at district hospitals closest to them.

The proportion of inpatients treated per medical personnel was negatively related to inefficiency scores, while the proportion of inpatients treated per nursing personnel was positively associated with inefficiency scores. It indicates that an increase in the ratio of inpatients to medical personnel would lead to a decrease in the inefficiency scores. In addition, the inefficiency scores would decline from a decrease in the proportion of inpatients to nursing personnel. This finding is similar to that obtained from a study conducted among eastern Ethiopian hospitals which also reported that the proportion of inpatients treated per medical doctor was negatively related to inefficiency scores [19]. The findings suggest a need for more efficient use of available doctors and nurses to provide health services. Thus, the efficiency of the DHs can be improved by ensuring a standard ratio of inpatients to medical and nurse personnel. The WHO recommended doctor to population ratio of 1 to 1000, and nursing personnel-to-population ratio of 4–1000 will be a helpful guide towards attaining the standard mix [36, 37]. A study conducted for the Hospital Association of South Africa reported that there were 25 public sector doctors per 100 000 people in South Africa [38]. This proportion translates to 16% of the global average ratio of 152 doctors per 100 000 population [39], and it is relatively low when compared to other BRICS member countries; India (70 per 100 000), Brazil (189 per 100 000), and China (194 per 100 000) [38].

Besides the WHO recommendations on the proportion of patients to medical and nursing personnel, it is essential to consider the skills mix of personnel, work-time, and healthcare needs of patients in different sections of the health facility. For instance, patients in intensive care units (ICUs) require frequent attention when compared to those in non-ICUs, such as the pre-operative unit. Studies have shown the significant impact of the above parameters on patient outcomes. A previous study showed that patients catered for by a substantial proportion of highly skilled (educated) nurses had a significant drop of 8% in the mortality rate [40]. Another research also showed that patients with greater needs, such as surgical patients, had better health outcomes when the proportion of skilled nursing personnel caring for them increased [41]. An additional personnel hour per patient days was found to be associated with a drop in admission rate in both intensive and non-intensive care units of the hospital [42]. In addition, an optimal proportion of patient-to-personnel ratio could assist in mitigating unnecessary waiting time usually experienced by patients [42]. In addition to the benefits to patients, an appropriate patient-to-health providers ratio could also prevent burnout and fatigue among medical and nursing personnel and thereby reducing occupational-related errors. Thus, optimal utilisation of trained nursing personnel to complement efforts of the relatively inadequate medical personnel in the public sector of the country could go a long way toward improving healthcare service delivery.

The negative statistically significant relationship between the expenditure per patient day equivalent and the inefficiency scores shows that a unit increase in the average spending per patient per day seen at the DHs would lead to a decrease in the inefficiency scores. The relationship suggests that to improve inefficient DHs, more funding should be pulled toward increasing the total health expenditure of the facilities, which will eventually translate to an increased average spending per patient per day. This increased expenditure per patient per day will ensure the availability of quality services and medical essentials such as drugs and other medical consumables needed by the patients. Alternatively, a frequent cost analysis of the health services provided by the district hospitals should be done to identify, where savings can be made without compromising the quality of health care.

However, findings from this study cannot be generalised as the situation of public health facilities in South Africa as district hospitals in other provinces, as well as different facility levels, such as primary and provincial healthcare facilities, were not included in the study. Future studies can look into the efficiency of these levels of health facilities and private health facilities and patients’ experience in the public health system, as well as the skill mix of healthcare providers. In addition, patients’ experience in either statistically efficient or inefficient facilities was unknown. Another limitation of this study was the unavailability of data on the severity of patient illness treated and case-mix. The national department of health should consider including information on the severity of illness treated in the health database. The public health system in South Africa was overstretched recently due to COVID-19 pandemic, resulting in a record spike in in-patient visits. Therefore, analysis and interpretation of recent health longitudinal data sets should be made cautiously.

## Conclusion

This study sheds light on the technical efficiency and factors influencing the technical efficiency of district hospitals in KwaZulu-Natal province, South Africa. The findings of this study showed that a significant proportion of the DHs was technically inefficient. The Tobit regression model identified catchment population, the proportion of inpatients treated per medical personnel, the proportion of inpatients treated per nursing personnel, and expenditure per patient day equivalent as factors influencing the efficiency of the DHs.

This study suggests that the technical efficiency of the DHs can be improved through; an improvement in the referral system and peoples’ health-seeking behaviour, and a standard mix of clinical staff for efficient service delivery. In addition, a periodic cost analysis of health services at the district hospitals with the view to saving costs and maintaining the quality of health care should be considered.

## Data Availability

The data set is available upon request to the corresponding author and permission from the department of health KwaZulu-Natal province.

## Abbreviations

BAS: Basic accounting system
BRICS: Brazil, Russia, India, China and South Africa
CRS: Constant returns to scale
CRSTE: Constant returns to scale technical efficiency
DEA: Data envelopment analysis
DH(s): District hospital(s)
DHIS: District health information system
nDOH: National department of health
PERSAL: Personnel salary system
SE: Scale efficiency
SSA: Sub-Saharan Africa
VRS: Variable returns to scale
VRSTE: Variable returns to scale technical efficiency
